# Supplementary material for: Machine Learning Accelerates Crystallization for Structure Determination
Source: Angew Chem Int Ed Engl. 2026 May 4;65(25):e1218503. doi: 10.1002/anie.1218503 (PMC13266958; doi:10.1002/anie.1218503)

checkCIF/PLATON report

Structure factors have been supplied for datablock(s) lcz052

THIS REPORT IS FOR GUIDANCE ONLY. IF USED AS PART OF A REVIEW PROCEDURE FOR PUBLICATION, IT SHOULD NOT REPLACE THE EXPERTISE OF AN EXPERIENCED CRYSTALLOGRAPHIC REFEREE.

No syntax errors found. CIF dictionary Interpreting this report

Datablock: lcz052

Bond precision:	C-C = 0.0135 A		Wavelength=1.54184
Cell:	a=12.6142(3)	b=22.0088(3)	c=13.4981(3)
	alpha=90	beta=116.760(3)	gamma=90
Temperature:	100 K		

	Calculated	Reported
Volume	3346.04(15)	3346.04(14)
Space group	P 21/n	P 1 21/n 1
Hall group	-P 2yn	-P 2yn
Moiety formula	C15 H3 Ag3 F18 N6, C12 H10 O	C15 H3 Ag3 F18 N6, C12 H10 O
Sum formula	C27 H13 Ag3 F18 N6 O	C27 H13 Ag3 F18 N6 O
Mr	1103.04	1103.04
Dx,g cm-3	2.190	2.190
Z	4	4
Mu (mm-1)	15.223	15.223
F000	2112.0	2112.0
F000'	2122.18	
h,k,lmax	15,27,17	15,27,16
Nref	7125	6767
Tmin,Tmax	0.116,0.138	0.570,1.000
Tmin'	0.001	

Correction method= # Reported T Limits: Tmin=0.570 Tmax=1.000
AbsCorr = MULTI-SCAN

Data completeness= 0.950 Theta(max)= 77.646

R(reflections)= 0.0797(5590) wR2(reflections)= 0.1836(6767)

S = 1.121 Npar= 447

The following ALERTS were generated. Each ALERT has the format
test-name_ALERT_alert-type_alert-level.
Click on the hyperlinks for more details of the test.

Alert level C

```
PLAT213_ALERT_2_C Atom F00K            has ADP max/min Ratio .....        3.6 prolat
PLAT213_ALERT_2_C Atom F00V            has ADP max/min Ratio .....        3.1 prolat
PLAT213_ALERT_2_C Atom F010            has ADP max/min Ratio .....        3.9 prolat
PLAT220_ALERT_2_C NonSolvent   Resd 1  F   Ueq(max)/Ueq(min) Range        3.3 Ratio
PLAT234_ALERT_4_C Large Hirshfeld Difference F015     --C01D     .       0.19 Ang.
PLAT234_ALERT_4_C Large Hirshfeld Difference C017     --C01G     .       0.18 Ang.
PLAT234_ALERT_4_C Large Hirshfeld Difference C019     --C014     .       0.17 Ang.
PLAT234_ALERT_4_C Large Hirshfeld Difference C01J     --C01C     .       0.21 Ang.
PLAT250_ALERT_2_C Large U3/U1 Ratio for <U(i,j)> Tensor(Resd    2)        2.6 Note
PLAT342_ALERT_3_C Low Bond Precision on  C-C Bonds ..............    0.01352 Ang.
PLAT362_ALERT_2_C Short  C(sp3)-C(sp2) Bond  C00T     - C01B     .       1.40 Ang.
PLAT362_ALERT_2_C Short  C(sp3)-C(sp2) Bond  C01D     - C00X     .       1.41 Ang.
PLAT906_ALERT_3_C Large K Value in the Analysis of Variance ......     18.725 Check
PLAT906_ALERT_3_C Large K Value in the Analysis of Variance ......      4.425 Check
PLAT911_ALERT_3_C Missing FCF Refl Between Thmin & STh/L=    0.600         46 Report
                0  2  0,   7  6  0,   4 11  1,  12  7  2,   1  0  3,   5 22  4,
               -5 13  7,   7  1  8,   8  4  8,  -6 12  8,  -6 13  8,  -5  0  9,
               -8  1  9,  -6 14  9,  -7 13 10,  -1  9 11,  -3 19 11,  -8  1 12,
                2  6 12,  -1  8 12,  -1  9 12,   0  9 12,   0 10 12,  -8 11 12,
               -8  1 13,  -8  2 13,  -8  1 14,  -4  6 14,  -5  0 15,  -7  1 15,
                   (   16 More Missing: see the .ckf listing file)
PLAT971_ALERT_2_C Check Calcd Resid. Dens.  1.47Ang From F00Q            1.83 eA-3
PLAT971_ALERT_2_C Check Calcd Resid. Dens.  0.36Ang From C00J            1.51 eA-3
PLAT977_ALERT_2_C Check Negative Difference Density on H00R      .      -0.31 eA-3
PLAT977_ALERT_2_C Check Negative Difference Density on H00U      .      -0.39 eA-3
PLAT977_ALERT_2_C Check Negative Difference Density on H01J      .      -0.43 eA-3
PLAT977_ALERT_2_C Check Negative Difference Density on H011      .      -0.60 eA-3
```

Alert level G

```
PLAT003_ALERT_2_G Number of Uiso or U(i,j)  Restrained non-H-Atoms          4 Report
PLAT083_ALERT_2_G SHELXL Second Parameter in WGHT  Unusually Large     108.86 Why ?
PLAT153_ALERT_1_G The s.u.'s on the Cell Axes   are Equal ..(Note)     0.0003 Ang.
PLAT186_ALERT_4_G The CIF-Embedded .res File Contains ISOR Records          1 Report
PLAT242_ALERT_2_G Low    'MainMol' Ueq as Compared to Neighbors of       C00Z Check
PLAT242_ALERT_2_G Low    'MainMol' Ueq as Compared to Neighbors of       C016 Check
PLAT242_ALERT_2_G Low    'MainMol' Ueq as Compared to Neighbors of       C018 Check
PLAT242_ALERT_2_G Low    'MainMol' Ueq as Compared to Neighbors of       C01A Check
PLAT242_ALERT_2_G Low    'MainMol' Ueq as Compared to Neighbors of       C01B Check
PLAT242_ALERT_2_G Low    'MainMol' Ueq as Compared to Neighbors of       C01D Check
PLAT434_ALERT_2_G Short Inter HL..HL Contact F004     ..F007     .       2.81 Ang.
                                                 -1+x,y,z  =      1_455 Check
```

```
PLAT720_ALERT_4_G Number of Unusual/Non-Standard Labels ..........         68 Note
              Ag01     Ag02     Ag03     F004     N005     C00J     C00R     H00R
              C00T     N00G     F006     F007     F009     F00A     F00B     F00D
              F00E     N00F     C00Y     C00W     H00W     C00S     N008     F00I
              F00K     O00L     F00M     F00N     F00O     F00P     F00Q     F00V
              C00Z     F010     F015     C016     C017     C018     C019     H019
              C014     C013     H013     C00U     H00U     C01F     C012     C01E
              H01E     C01I     H01I     C01J     H01J     C01C     H01C     C01A
              C01B     C01D     C01G     H01A     H01B     H01D     C01H     N00C
              N00H     C00X     C011     H011
PLAT860_ALERT_3_G Number of Least-Squares Restraints ............         24 Note
PLAT883_ALERT_1_G Absent Datum for _atom_sites_solution_primary ..     Please Do !
PLAT912_ALERT_4_G Missing # of FCF Reflections Above STh/L=  0.600        304 Note
PLAT913_ALERT_3_G Missing # of Very Strong Reflections in FCF ....          1 Note
                1  0  3,
PLAT933_ALERT_2_G Number of HKL-OMIT Records in Embedded .res File         27 Note
               -8  1  9,  -8  1 12,  -8  1 13,  -8  1 14,  -8  2 13,  -7  1 15,
               -7  2 15,  -6  0 16,  -6  1 16,  -6  2 16,  -5  1 16,  -5  2 16,
               -5  3 16,  -5  5 16,  -4  4 16,  -4  5 16,  -4  7 15,  -3  3 16,
               -3  4 16,  -3  5 16,  -3  6 15,  -3  7 15,  -2  6 15,  -1  5 15,
                0  9 12,   2  6 12,   7  1  8,
PLAT941_ALERT_3_G Average HKL Measurement Multiplicity ..........        2.5 Low
PLAT969_ALERT_5_G The 'Henn et al.' R-Factor-gap value ..........      3.123 Note
              Predicted wR2: Based on SigI**2  5.88 or SHELX Weight 16.37
PLAT978_ALERT_2_G Number C-C Bonds with Positive Residual Density.          1 Info
```

```
   0 ALERT level A = Most likely a serious problem - resolve or explain
   0 ALERT level B = A potentially serious problem, consider carefully
  21 ALERT level C = Check. Ensure it is not caused by an omission or oversight
  20 ALERT level G = General information/check it is not something unexpected

   2 ALERT type 1 CIF construction/syntax error, inconsistent or missing data
  24 ALERT type 2 Indicator that the structure model may be wrong or deficient
   7 ALERT type 3 Indicator that the structure quality may be low
   7 ALERT type 4 Improvement, methodology, query or suggestion
   1 ALERT type 5 Informative message, check
```

Validation response form

Please find below a validation response form (VRF) that can be filled in and pasted into your CIF.

```
# start Validation Reply Form
_vrf_PLAT213_lcz052
;
PROBLEM: Atom F00K            has ADP max/min Ratio .....        3.6 prolat
RESPONSE: ...
;
_vrf_PLAT220_lcz052
;
PROBLEM: NonSolvent   Resd 1  F   Ueq(max)/Ueq(min) Range        3.3 Ratio
RESPONSE: ...
;
_vrf_PLAT234_lcz052
;
PROBLEM: Large Hirshfeld Difference F015     --C01D     .       0.19 Ang.
```

```
RESPONSE: ...
;
_vrf_PLAT250_lcz052
;
PROBLEM: Large U3/U1 Ratio for <U(i,j)> Tensor(Resd    2)        2.6 Note
RESPONSE: ...
;
_vrf_PLAT342_lcz052
;
PROBLEM: Low Bond Precision on  C-C Bonds ..............    0.01352 Ang.
RESPONSE: ...
;
_vrf_PLAT362_lcz052
;
PROBLEM: Short  C(sp3)-C(sp2) Bond  C00T     - C01B     .       1.40 Ang.
RESPONSE: ...
;
_vrf_PLAT906_lcz052
;
PROBLEM: Large K Value in the Analysis of Variance ......     18.725 Check
RESPONSE: ...
;
_vrf_PLAT911_lcz052
;
PROBLEM: Missing FCF Refl Between Thmin & STh/L=    0.600         46 Report
RESPONSE: ...
;
_vrf_PLAT971_lcz052
;
PROBLEM: Check Calcd Resid. Dens.  1.47Ang From F00Q           1.83 eA-3
RESPONSE: ...
;
_vrf_PLAT977_lcz052
;
PROBLEM: Check Negative Difference Density on H00R      .      -0.31 eA-3
RESPONSE: ...
;
# end Validation Reply Form
```

It is advisable to attempt to resolve as many as possible of the alerts in all categories. Often the minor alerts point to easily fixed oversights, errors and omissions in your CIF or refinement strategy, so attention to these fine details can be worthwhile. It is up to the individual to critically assess their own results and, if necessary, seek expert advice.

PLATON version of 17/09/2025; check.def file version of 17/09/2025

---

duplicate check

No duplication found

Datablock lcz052 - ellipsoid plot

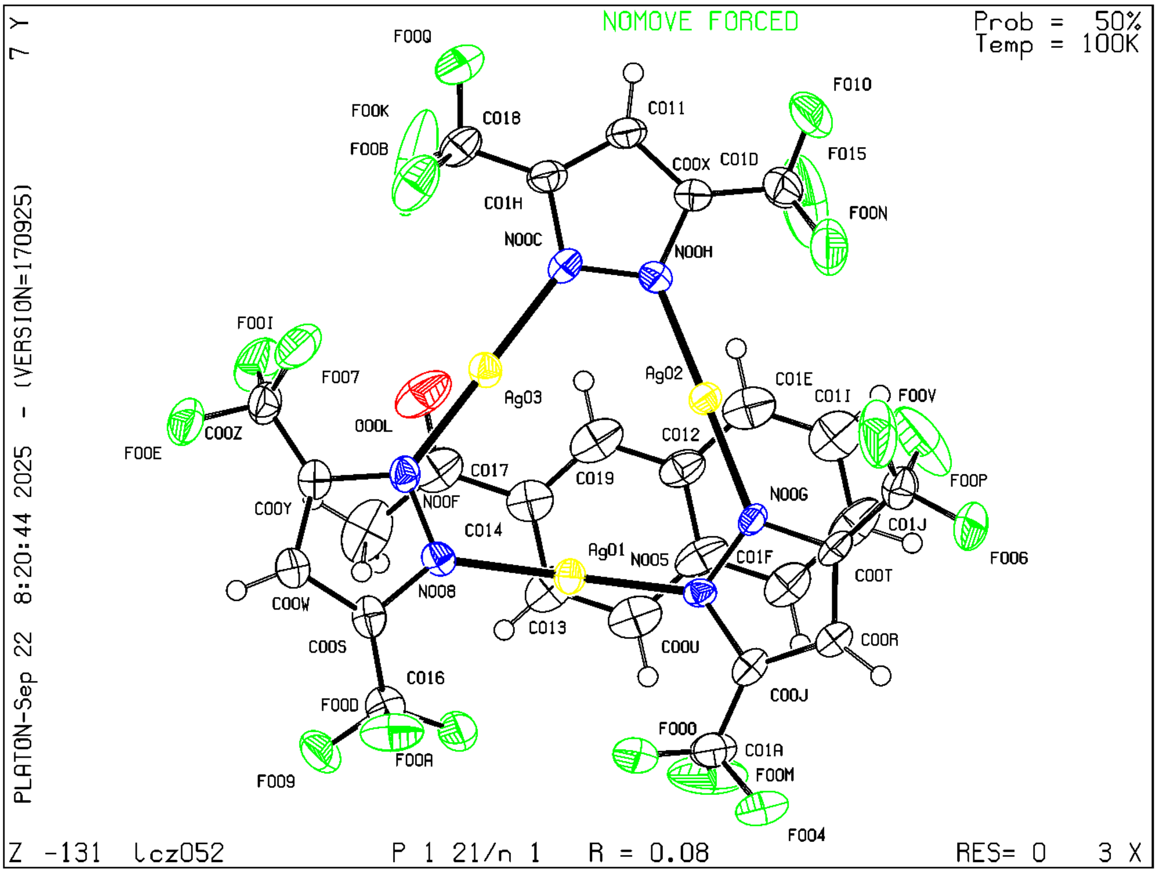

Supplement: Supplementary file 2 — Supporting File 2: anie72462‐sup‐0002‐DataFile.zip. [file ANIE-65-e1218503-s002.zip › supplementary_materials_crystallographic_data submit.zip!/cif_and_checkcif(1-114)/Ag3Pz3_52/Ag3Pz3_52_checkcif.pdf]
